# Evaluation of the Clinical Impact of a Smartphone Application for Cataract Detection

**DOI:** 10.7759/cureus.71467

**Published:** 2024-10-14

**Authors:** Siddharam S Janti, Rohit Saluja, Nivedita Tiwari, Raghavendra Rao Kolavai, Kalpana Mali, Abhishek J Arora, Amita Johar, Durgesh Prasad Sahoo, Eereti Sahithi

**Affiliations:** 1 Ophthalmology, All India Institute of Medical Sciences, Bibinagar, Bibinagar, IND; 2 Biochemistry, All India Institute of Medical Sciences, Bibinagar, Bibinagar, IND; 3 Pharmacology, All India Institute of Medical Sciences, Bibinagar, Bibinagar, IND; 4 Radiodiagnosis, All India Institute of Medical Sciences, Bibinagar, Bibinagar, IND; 5 Artificial Intelligence and Machine Learning, Samskruti College of Engineering and Technology, Hyderabad, IND; 6 Community and Family Medicine, All India Institute of Medical Sciences, Bibinagar, Bibinagar, IND

**Keywords:** artificial intelligence, cataract, deep learning, digital health, smart phone, telemedicine

## Abstract

Background

Approximately 10 million people in India suffer from bilateral blindness, with cataracts accounting for roughly 70% of these cases. However, there is a severe scarcity of ophthalmologists in India (12,000 across the country), which makes routine cataract screening very difficult, particularly in rural areas. To tackle this problem, we investigated the use of an artificial intelligence (AI)-based application for cataract screening at All India Institute of Medical Sciences (AIIMS), Bibinagar, that can be used by nursing officers and other healthcare professionals as a primary screening tool. Ophthalmologists from AIIMS Bibinagar additionally validate the results of this application.

Purpose

The aim of this study was to assess the clinical performance of a smartphone-based cataract screening application that uses an AI module to identify cataracts in photos taken with the device's camera. The study compared the application’s results with diagnoses made by ophthalmologists using a slit lamp.

Methods

At AIIMS Bibinagar, 495 patients participated in a prospective clinical trial. The AI-based screening solution examined smartphone images that were taken in accordance with a set protocol to identify whether cataracts were present. The results of the application were then compared with the diagnoses made by ophthalmologists based on slit-lamp tests.

Results

The study included 990 eye images. The AI screening application demonstrated an overall accuracy of 90.01% for cataract detection. Specific metrics include a sensitivity of 89.50%, specificity of 89.73%, precision of 91.43%, and an F1 score of 90.36%. The positive predictive value (PPV) was approximately 91.3%, based on 485 true positives and 46 false positives. The negative predictive value (NPV) was approximately 87.6%, based on 402 true negatives and 57 false negatives.

Conclusions

The smartphone-based cataract screening application proves to be an effective tool for community-level cataract screening in remote areas where access to expensive equipment and specialized ophthalmic care is limited. Its high accuracy and efficiency make it a valuable option for low-resource settings and suitable for home screening, particularly in the post-COVID era.

## Introduction

Blindness caused by cataracts has been a significant global health issue for decades, though it was not always recognized. Today, cataract-related blindness is a well-understood public health challenge, particularly in rural areas with limited access to eye care services [1,2]. In 2020, an estimated 2.2 million people in low- and middle-income countries were affected by cataract-induced blindness [3]. Without timely intervention, this number is projected to rise to 3.6 million by 2050 [4]. Traditionally, cataracts are diagnosed by ophthalmologists using slit lamps. However, conducting mass community screenings in remote or underserved areas is challenging with conventional methods. Alternative techniques such as handheld portable slit lamps, smartphone attachments, fundus photography, and slit lamp images have been explored [5], but these approaches often require costly equipment and specialized skills.

The primary objective of this study was to comprehensively evaluate the clinical impact of a smartphone application designed for the first level of cataract screening. This evaluation focused on assessing the performance and credibility of the application as a reliable and effective tool in a clinical setting. By systematically analyzing user outcomes, diagnostic accuracy, and overall usability, the study aimed to determine the potential of the application to enhance clinical practice and improve patient care.

There is a pressing need for innovative solutions to address these limitations and improve cataract detection, especially in low-resource settings [6]. Computing and artificial intelligence (AI), including machine learning (ML) and deep learning (DL), hold promise for expanding the reach and efficiency of digital ophthalmology [7]. AI has already shown potential in diagnosing various ophthalmic conditions, such as diabetic retinopathy, glaucoma, and macular degeneration [8-12]. An AI-based analysis of images captured with smartphones without the need for additional external hardware can be particularly advantageous. Smartphones are widely available and cost-effective, making them suitable for remote areas. Researchers have developed solutions that use luminance-based eye image analysis to detect cataracts from smartphone images [13-14]. Similarly, a novel AI-based model has been created to detect cataracts using images captured by a smartphone camera. This model utilizes images taken with a smartphone’s flashlight and employs DL algorithms to identify patterns indicative of cataracts.

## Materials and methods

**Figure 1:**
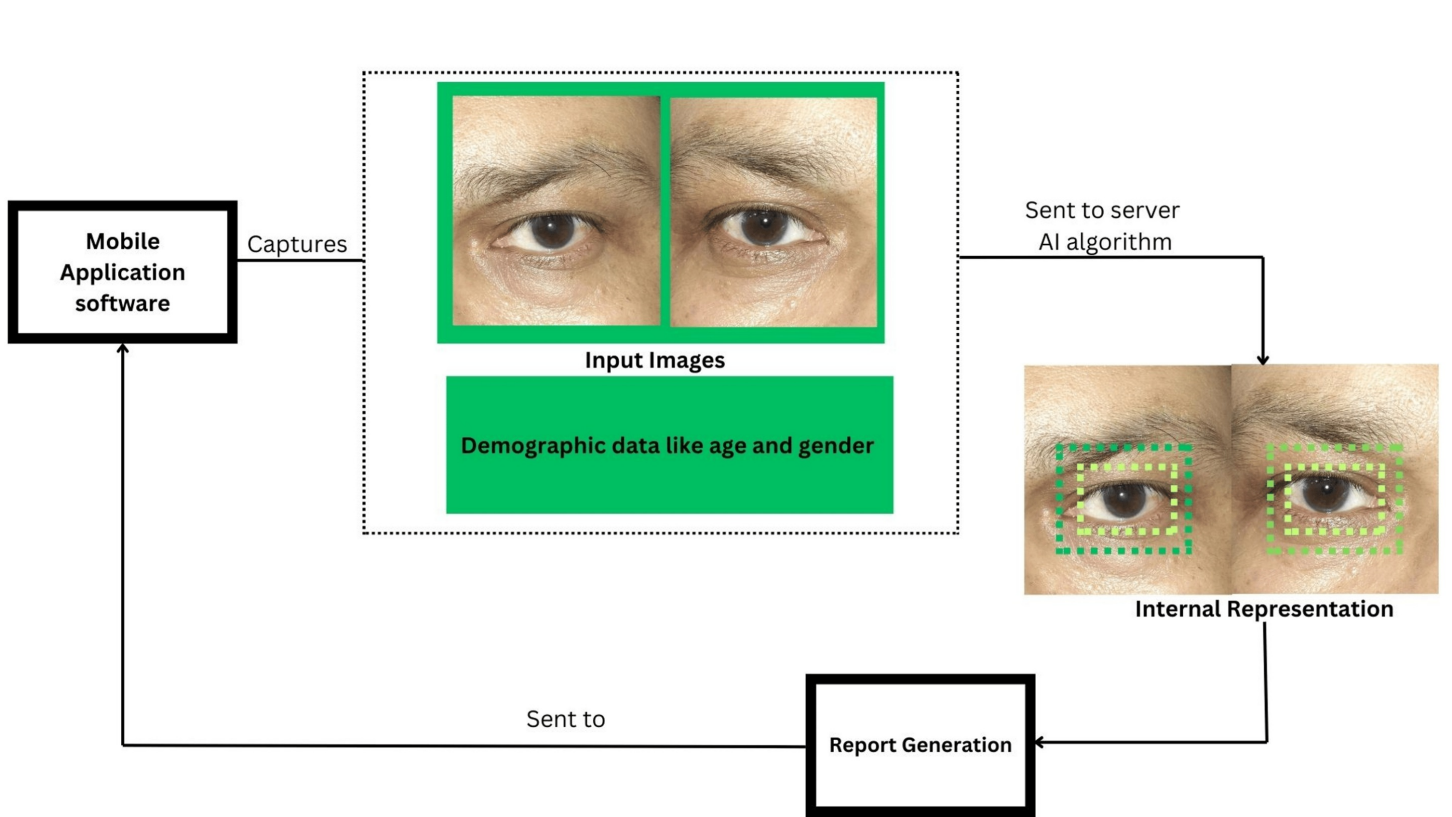
Application screening flow: the working model of the cataract screening solution is depicted in a flowchart.

A prospective, observational diagnostic accuracy study was conducted to evaluate the accuracy of the smartphone eye screening solution for cataract detection. It took place at the AIIMS Bibinagar, Hyderabad, Telangana, India, over a six-month period from April 2024 to July 2024. The study protocol received approval from the ethics committee and the institutional review board of AIIMS Bibinagar (Registration Number: ECR/1596/Inst/TG/2021). This trial is registered with Clinical Trials (CTRI/2024/04/065473). A total of 495 patients were recruited from the eye clinic at AIIMS Bibinagar. Written informed consent was obtained from each patient to capture their eye images for AI analysis. Demographic information, including age and gender, along with images of both eyes, was collected using a Redmi 9A smartphone equipped with a 13 MP f/2.2 main camera and LED flash. The images were captured by two trained optometrists. These images were then uploaded to the app-based screening solution (Figure 1), which generated a unique identification number (ID) for each participant. The AI system analyzed each eye image to predict the presence of a cataract or a normal lens, with intraocular lenses (IOLs) also classified as normal. Cataracts were further categorized as positive or negative. Subsequently, each patient underwent an examination by three principal investigators, all experienced ophthalmologists at AIIMS Bibinagar, using slit lamps with appropriate illumination and eye positioning. The ophthalmologists' assessments were documented and later digitized for comparison with the AI-generated predictions.

The primary outcome measure was the accuracy of the eye screening solution in distinguishing between normal lenses and cataracts. Secondary outcome measures included the sensitivity, specificity, precision, and F1 score of the eye screening solution, as well as the accuracy of the AI-based grading. These metrics are essential for evaluating the performance of classification models in machine learning. Sensitivity is particularly important in contexts where minimizing false negatives is critical, such as in medical diagnoses, while specificity is vital for reducing the occurrence of false positives.

Sample size

Using the equation provided, a sample size of at least 495 patients (990 eyes) was required to measure the diagnostic accuracy between the application and senior ophthalmologists. Of the 990 eyes, 542 were detected as positive for cataracts. The sample size calculation was based on the expected accuracy of 90% in the AI arm and 95% in the senior consultant’s arm, with an 80% statistical power and a 5% statistical significance level. The sensitivity of the study was 96.7%, with a precision of 3%. These estimated values of sensitivity and specificity are derived from the findings of a pilot study conducted at Sharp Sight Hospital, involving 100 patients (50 in each category). This pilot study aimed to validate the diagnostic accuracy between ophthalmologists and the screening app. Assuming a 95% CI and a cataract prevalence of 47.5%, the sample size was calculated using the formula: \[ N = \frac{Z2_{(1-\alpha/2)} \times Se \times (1 - Se)}{d2} \] where N represents the sample size, which is the number of observations or data points needed for a particular study or analysis. Z2_{(1-\alpha/2)} iis the Z-score squared, corresponding to the confidence interval. The Z-score represents the number of standard deviations a data point is from the mean. The subscript (1−α/2)(1-\alpha/2)(1−α/2) indicates the Z-score associated with the desired confidence level (for example, a 95% CI). Se stands for sensitivity, which measures the proportion of true positives that are correctly identified by the test or model. Sensitivity reflects the ability of the AI-based system to correctly detect positive cases. (1 - Se) represents the proportion of false negatives, or the probability of a true positive being missed by the test or model, and d2 is the square of the precision, which defines the acceptable margin of error or the allowable deviation from the true value in the study.

Inclusion criteria

After a thorough explanation, all patients who were willing to participate in the study were selected. The study included patients who were over 45 years of age, and participants of both genders, including males and females, were considered for inclusion.

Exclusion criteria

**Figure 2:**
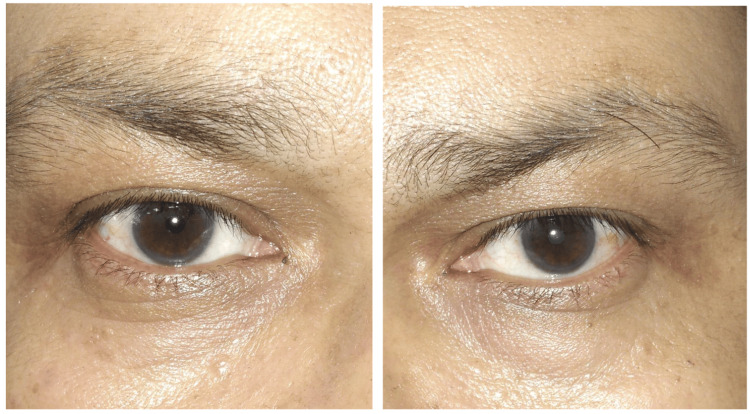
Sample left and right eye images: images captured for both the left and right eye of a participant, sent to AI for cataract prediction.

Patients found to be critically ill after the examination, and those who were not willing to participate in the study, were excluded. After informing the study, a trained technician collected the images of the patients with decreased vision using a smartphone and uploaded the data to the cloud system. Then, patients were referred to the Ophthalmology OPD at AIIMS Bibinagar. Here, patients were examined by trained ophthalmologists via Slit Lamp Examination at AIIMS Bibinagar Ophthalmology Department. After dilation of pupils using Tropicamide+Phenylephrine or plain Tropicamide eye drops, detailed anterior and posterior segment examinations of the eye were conducted. If a cataract was found, it was categorized using the LCOS III system. All findings were detailed in the OPD sheet. In the end, two types of data were collected and stored: photographic images of the eye (both left and right eye) (Figure 2) and ophthalmologist feedback on patient reports with diagnosis. The images that were transferred to the cloud system were analyzed by the personnel at the collection center itself. All the images were collated as per unique patient ID generated onsite and ingested into the cloud-based platform.

Technical analysis

The screening solution uses an ensemble of deep learning models with convolutional neural networks (CNN) and other image processing techniques such as computer vision. CNN is a type of artificial neural network that assists in image/object recognition. To learn spatial hierarchies of features from the images or objects, the CNN system is designed to automatically and adaptively learn patterns from low-level to higher-level. With the features extracted from the CNN layers as inputs, these features are sent to a dense layer classifier. Based on these inputs, the classifier generates the corresponding output probability. The screening system is already trained on thousands of eye images so that it can find patterns similar to those in cataract-affected eyes. Essentially, it takes an eye image as an input, finds the required eye area, and then predicts the diagnosis (normal lens vs. cataract) based on lens opacity, cloudiness, and iris shadow. The model is trained using PyTorch [15], TorchVision, and PyTorch Lightning [16]. The eye images are taken in a special way that they represent only the corresponding (left or right) eye and no other part. The flowchart in Figure 1 illustrates the operational model of the cataract screening solution.

Statistical analysis

Descriptive statistics, including mean, standard deviation, and frequency, were used to analyze demographic data such as age and gender. The IQR was calculated for continuous variables, with IQR bounds used to identify outliers. Statistical measures such as accuracy, sensitivity, specificity, positive predictive value (PPV), and negative predictive value (NPV), along with their Clopper-Pearson 95% confidence intervals, were computed. Receiver Operating Characteristic (ROC) curves and the area under the curve (AUC) were used to assess the model’s predictive ability across various thresholds. Data analysis was performed using Python packages such as Pandas, Scikit-learn [16], SciPy [17], and NumPy [18]. Histograms, curves, and other figures were generated using Seaborn [19] and Matplotlib [20].

## Results

The study aimed to evaluate the diagnostic accuracy of an AI-based screening solution for cataract detection by comparing its performance to that of senior ophthalmologists (Table 1). The overall accuracy of the AI model was determined to be 90.01% with a 95% CI ranging from 87.98% to 92.18%. This level of accuracy indicates robust performance, but a detailed analysis through the confusion matrix provides deeper insights into the model’s strengths and limitations.

**Table 1:** AI predictions vs. ophthalmologist diagnosis.

Class Type	AI Predictions	Ophthalmologist Diagnosis
Cataract Positive (Mature or Immature)	531	542
Cataract Negative (Normal and Intraocular Lens)	459	448

The confusion matrix (Table 2) revealed that the AI correctly identified 485 cases as cataract-positive (true positives) and 402 cases as normal (true negatives). However, there were 46 instances where the AI incorrectly identified normal eyes as cataract-positive (false positives), and 57 instances where cataract-positive eyes were mistakenly identified as normal (false negatives). This discrepancy of 103 images highlights the areas where the AI's predictions diverged from those of the ophthalmologists, underscoring the importance of continuous model refinement.

**Table 2:** The confusion matrix results. The table presents the confusion matrix, highlighting that 46 images were false positives and 57 were false negatives. There was a discrepancy in the diagnosis of 103 images between the ophthalmologists and the AI predictions.

		Ophthalmologists Diagnosis		Total
		Positive	Negative	
AI Predictions	Positive	485 (True Positive)	46 (False Positive)	531
	Negative	57 (False Negative)	402 (True Negative)	459
Total		542	448	990

To further understand the AI’s diagnostic capabilities, sensitivity and specificity were calculated (Table 3). The sensitivity, or the model’s ability to correctly identify cataract-positive cases, was approximately 89.50%. This means that the AI successfully detected cataracts in nearly 90% of the cases where the condition was present. On the other hand, the specificity, which measures the model’s ability to correctly identify normal cases, was approximately 89.73%. This indicates that the AI was equally proficient at identifying eyes without cataracts.

**Table 3:** AI-system based test evaluation.

Parameter	Estimate	Lower-Upper 95% CI
Sensitivity	89.48%	(86.62, 91.79)
Specificity	89.73%	(86.58, 92.21)
Positive Predictive Value	91.34%	(88.64, 93.44)
Negative Predictive Value	87.58%	(84.25, 90.29)
Diagnostic Accuracy	89.60%	(87.54, 91.35)
Likelihood ratio of a Positive Test	8.715	(8.347-9.099)
Likelihood ratio of a Negative Test	0.1172	(0.1132-0.1214)

The overall accuracy of 90.01% reflects the model’s effectiveness in correctly identifying both cataract-positive and normal cases. However, the identified false positives and false negatives highlight the need for further improvements to minimize diagnostic errors. The detailed evaluation of sensitivity and specificity provides a comprehensive understanding of the AI’s performance, which is crucial for its potential deployment in clinical settings. This analysis serves as a foundation for future research aimed at enhancing the accuracy and reliability of AI in medical diagnostics.

A total of 495 patients were enrolled in the study, resulting in 990 eye images (comprising both right and left eye images). Among these, doctors identified 542 images as positive for cataracts. In comparison, the AI module detected 531 cataract-positive images. The AI system is designed to classify cataracts into two categories: positive or negative, and it also identifies clear lenses and intraocular lenses (IOLs) as normal. Further categorization of cataracts into immature and mature stages is detailed in Table 1.

Other analysis

**Figure 3:**
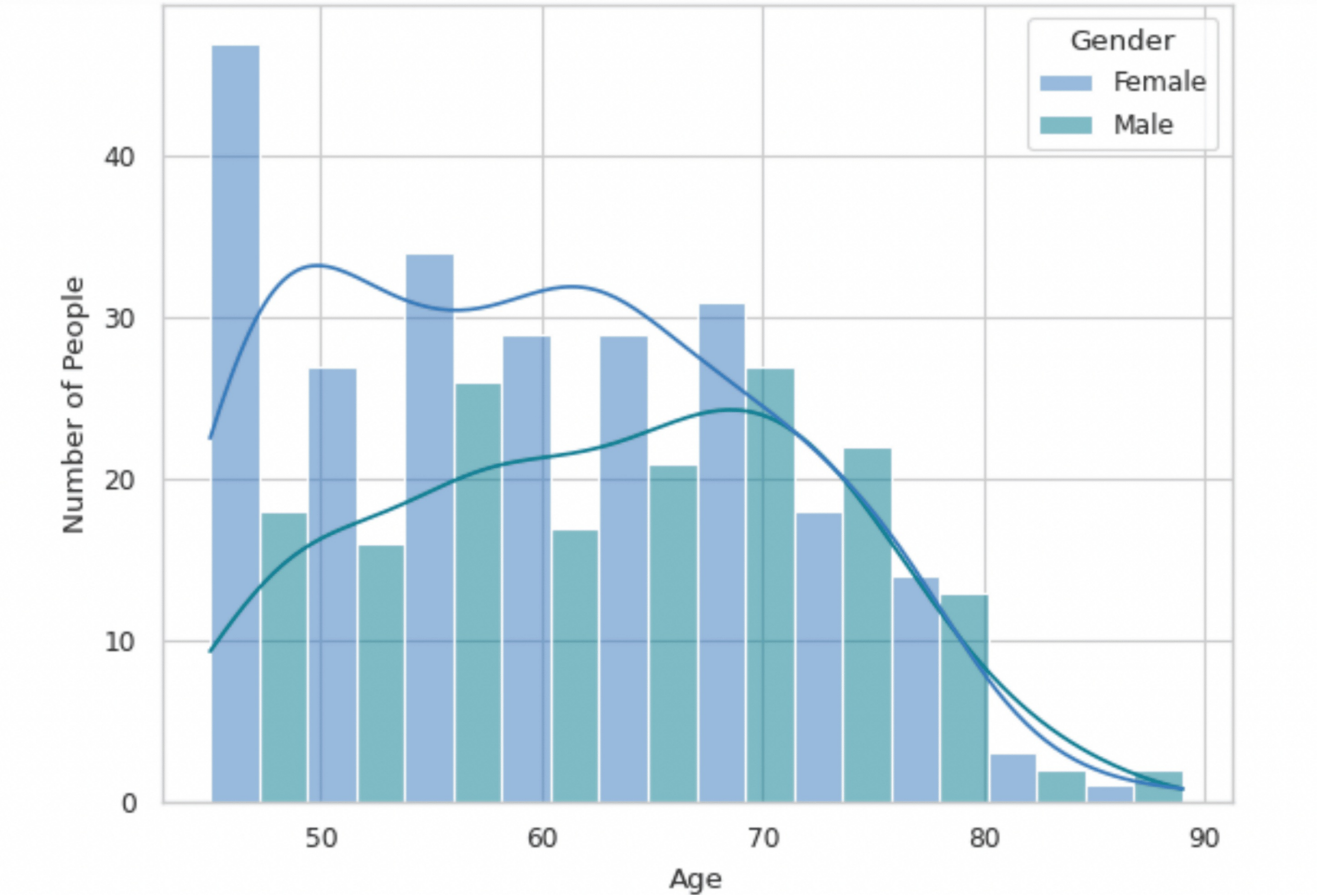
Age distribution by gender. The graph shows the distribution of people by age and gender, with bars representing the number of males and females across different age groups. The highest concentration is seen in the 50s, especially among females, with the numbers gradually decreasing in older age groups. The overlaid lines represent the density distribution, showing a higher prevalence of females in younger and middle-age groups, while males are more evenly distributed.

Among the 495 patients recruited, there were 325 females and 170 males. The mean age of the entire group is 61.2 years, with males having a mean age of 63.04 years and females having a mean age of 59.96 years. The IQR for the combined age data is 17.0 years, with a range of 52.0-69.0 years. For males, the IQR is 15.0 years, with a range of 56.0-71.0 years, and for females, the IQR is 18.0 years, with a range of 50.0-68.0 years. There were no outliers detected. The demographic distribution of age by gender is illustrated in Figure 3. 

**Figure 4:**
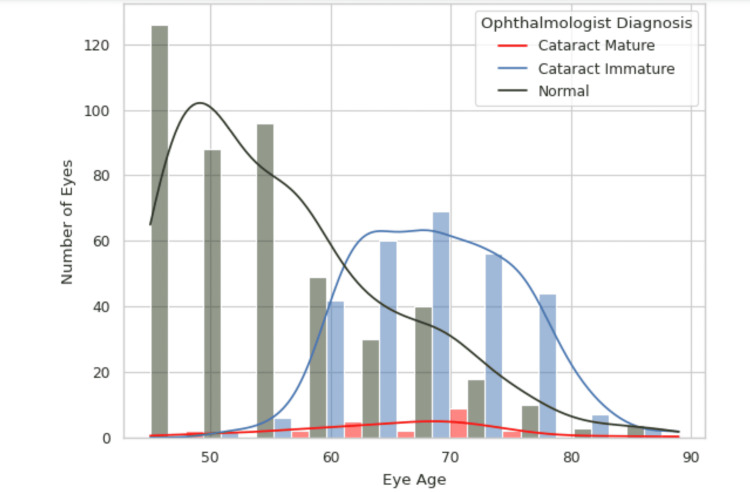
Eye age distribution by cataract cases (ophthalmologist diagnosis). The graph illustrates the distribution of eye conditions (normal, immature cataracts, and mature cataracts) across different age groups. The majority of eyes are normal, particularly in younger age groups, with a notable peak around age 50. As age increases, the prevalence of immature cataracts rises, peaking in the 70s, while mature cataracts remain relatively low across all age groups. In the study, the detection of overall positive cataract cases is considered.

As age advances, the likelihood of developing cataracts becomes more pronounced. Within the age bracket of 40 to 45 years, the incidence of cataract cases is relatively low, indicating that cataract formation is less common during these years. However, as individuals approach the age of 45 and beyond, the risk of cataract development rises substantially. This increase is due to the natural aging process, where changes in the lens of the eye become more prevalent, leading to a higher probability of clouding and the eventual formation of cataracts. Consequently, while cataracts can occur in middle-aged adults, they are far more common and pose a greater risk as individuals grow older, particularly as they reach and exceed the age of 60 (Figure 4). 

**Figure 5:**
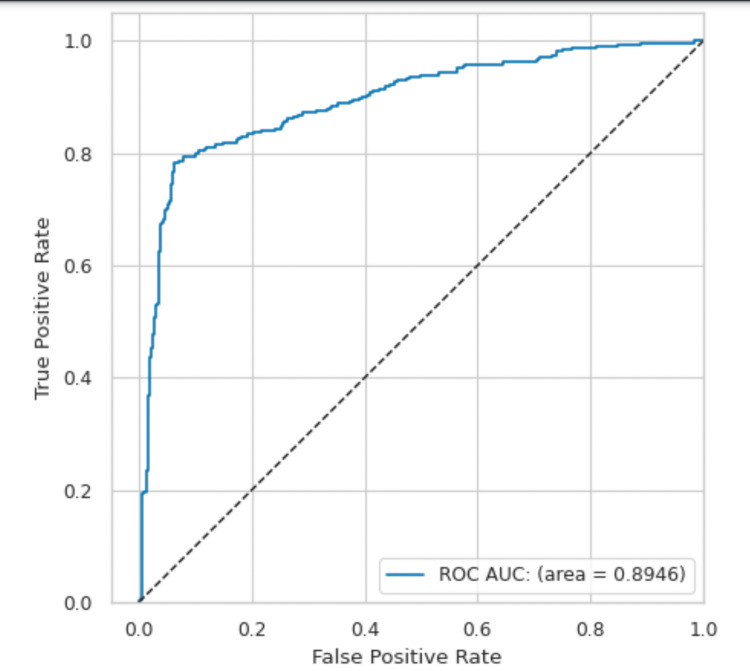
The receiver operating characteristic (ROC) curve of the AI module with an area under the curve (AUC) of 0.8946. The ROC curve visually represents the trade-off between sensitivity and specificity. A convex ROC curve indicates good model performance, whereas a curve close to the diagonal line (less convex) suggests poorer performance. The AUC for the cataract prediction module falls within the good category, reflecting its effectiveness in distinguishing between cataract-positive and normal cases.

The ROC curve visually represents the trade-off between a model's sensitivity and specificity. A convex curve above the diagonal line indicates good model performance, balancing true positive and true negative rates effectively. If the curve is close to the diagonal, it suggests poor performance, with the model only slightly better than random guessing. The curve's shape and position thus reflect the model's ability to distinguish between positive and negative cases (Figure 5).

## Discussion

In India, over 10 million people suffer from bilateral blindness, with cataracts being the leading cause in approximately 70% of cases. Early detection and surgical removal can restore vision, but a significant challenge exists due to the shortage of ophthalmologists-only 12,000 in the country. This results in a ratio of roughly one ophthalmologist per 250,000 people in rural areas, making regular cataract screening difficult and leading to many cases going undetected, resulting in preventable blindness.

In contrast, a similar application, the e-parvai app, demonstrated high sensitivity but lower specificity (25%) and less effective performance for mature cataract [21]. It also required additional installation. The AI-based application discussed here overcomes these limitations with improved specificity (89.87%) and negative predictive value (93.29%), reducing the need for patient visits to tertiary care centers and thus decreasing the healthcare burden. Although the current study’s sample size was limited, particularly for advanced cataracts, further research with larger samples is needed to refine and validate the screening of mature cataracts.

Gao X et al. [22] proposed a system in 2015 that combined convolutional neural networks (CNN), recurrent neural networks (RNN), and support vector regression (SVR) for grading cataracts using slit-lamp images. This system achieved a 70.7% agreement ratio for detecting referable cataracts. In contrast, Li W et al. [23] developed a proprietary model, Visionome, to identify and annotate cataract and other anterior segment pathologies from slit-lamp images. Visionome's performance was comparable to that of experienced ophthalmologists, with an accuracy range of 79.47% to 99.22%. Wu X et al. [24] designed a ResNet deep learning algorithm that differentiated between cataractous lenses, intraocular lenses (IOLs), and normal lenses, achieving an AUC greater than 0.91. Their telemedicine platform allowed patients to submit smartphone photos of their eyes, with the algorithm determining if a referral to a healthcare facility was necessary.

Currently, cataracts are diagnosed clinically using slit lamps, necessitating face-to-face consultations. This approach poses significant challenges in developing countries and rural areas due to limited accessibility. An AI-assisted telemedicine platform for preliminary cataract diagnosis could help bridge this gap and alleviate the healthcare burden.

AI is revolutionizing various fields, including healthcare. In ophthalmology, AI technologies such as ML and DL offer promising solutions for cataract detection. Traditional methods, such as slit lamps and fundus cameras, are expensive and require trained professionals, making them less feasible for widespread use. With the advent of AI, it is now possible to analyze digital images captured by smartphones, which are more accessible and cost-effective. The current study evaluates an AI-based smartphone application for cataract detection. This application, which functions through a standard smartphone without requiring additional installations, achieved an accuracy of 90.08%, with a sensitivity of 90.38% and a specificity of 89.87%. The area under the ROC curve indicates strong performance, making it a valuable screening tool.

This smartphone-based screening tool represents a significant advancement over existing methods, which primarily rely on slit-lamp and fundus images. It offers a practical solution for digital tele-screening, addressing diagnostic, logistical, and operational challenges. The application can be easily installed on most smartphones, performs screening in under two minutes, and provides electronic reports. It is well-suited for community screening camps, primary healthcare centers, and home use. In the post-COVID era, where reducing hospital visits is a priority, this tool offers an ideal alternative for early cataract detection, facilitating tele-ophthalmology and opening avenues for further educational and research initiatives.

This study encountered a few limitations. First, there was inconsistent adherence to optimal zoom and lighting conditions during image capture, which might have affected the quality and accuracy of the images used for AI analysis. Such variations can influence the performance of the AI model. Additionally, the model occasionally misclassified clear lenses and intraocular lenses as normal, indicating that the AI algorithm may require further development to better differentiate between these lens types and true cataract conditions. Addressing these limitations in future research could enhance the accuracy and robustness of AI-based cataract detection systems.

## Conclusions

Cataract-induced blindness remains a major global health concern, particularly affecting rural and underserved areas with limited access to eye care. Conventional diagnostic methods, including slit lamps, pose significant challenges for mass screenings in remote locations due to their cost and the need for specialized skills. Advances in technology, particularly through the use of smartphones and AI, present promising alternatives. AI models leveraging deep learning algorithms have shown the potential to enhance cataract detection by analyzing images captured with smartphone cameras. This approach benefits from the widespread availability and affordability of smartphones, making it feasible.

## Figures and Tables

**Figure 6:**
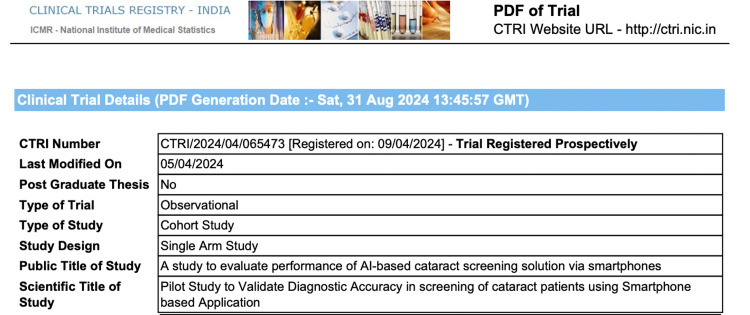
CTRI registration. CTRI: Clinical Trials Registry India.
